# Interactome analyses revealed that the U1 snRNP machinery overlaps extensively with the RNAP II machinery and contains multiple ALS/SMA-causative proteins

**DOI:** 10.1038/s41598-018-27136-3

**Published:** 2018-06-08

**Authors:** Binkai Chi, Jeremy D. O’Connell, Tomohiro Yamazaki, Jaya Gangopadhyay, Steven P. Gygi, Robin Reed

**Affiliations:** 1 Department of Cell Biology, Harvard Medical School, 240 Longwood Ave, Boston, MA 02115 USA; 2 Department of Microbiology and Immunology, Stanford University School of Medicine, 291 Campus Drive, Stanford, CA 94305 USA

## Abstract

Mutations in multiple RNA/DNA binding proteins cause Amyotrophic Lateral Sclerosis (ALS). Included among these are the three members of the FET family (FUS, EWSR1 and TAF15) and the structurally similar MATR3. Here, we characterized the interactomes of these four proteins, revealing that they largely have unique interactors, but share in common an association with U1 snRNP. The latter observation led us to analyze the interactome of the U1 snRNP machinery. Surprisingly, this analysis revealed the interactome contains ~220 components, and of these, >200 are shared with the RNA polymerase II (RNAP II) machinery. Among the shared components are multiple ALS and Spinal muscular Atrophy (SMA)-causative proteins and numerous discrete complexes, including the SMN complex, transcription factor complexes, and RNA processing complexes. Together, our data indicate that the RNAP II/U1 snRNP machinery functions in a wide variety of molecular pathways, and these pathways are candidates for playing roles in ALS/SMA pathogenesis.

## Introduction

The neurodegenerative disease Amyotrophic Lateral Sclerosis (ALS) has no known treatment, and elucidation of disease mechanisms is urgently needed. This problem has been especially daunting, as mutations in greater than 30 genes are ALS-causative, and these genes function in numerous cellular pathways^1^. These include mitophagy, autophagy, cytoskeletal dynamics, vesicle transport, DNA damage repair, RNA dysfunction, apoptosis, and protein aggregation^2–6^. The discovery that mutations in two RNA/DNA binding proteins, FUS and TARDBP, are ALS-causative first raised the possibility that dysfunction of RNA-related processes plays a role in the disease^7–11^. This hypothesis gained traction when additional ALS-causative RNA/DNA binding proteins (EWSR1, TAF15, HNRNPA1, HNRNPA2B1, MATR3 and TIA1) were identified^12–16^. At present, however, the roles of these proteins in ALS pathogenesis are not known.

FUS, EWSR1 and TAF15 constitute the FET family of structurally related proteins^17,18^. They share in common RNA binding motifs and low complexity domains. Similar to the FET family members, MATR3 also contains both types of domains^19^. Although ample evidence exists that all four of these ALS-causative proteins function in transcription and splicing, much less is known about how their functions are distinguished from one another in these processes. We recently found that the four ALS-causative proteins associate with the RNAP II machinery and that several other ALS-causative proteins, including HNRNPA1^20^, HNRNPA2B1^20^, TIA1^16^ and VCP^21^, do as well (BC *et al*., submitted). Moreover, multiple proteins that are mutated in the childhood motor neuron disease cause Spinal Muscular Atrophy (SMA) associate with the RNAP II machinery, including SMN1, EXOSC8^22^, HSPB1^23,24^ and two components (ASCC1 and TRIP4)^25,26^ of the ASC-1 transcriptional co-activator (BC *et al*., submitted). To investigate the roles of ALS-causative proteins within the RNAP II machinery, we used CRISPR to knock out the 3 FET family members or MATR3 in HeLa cells and then characterized the RNAP machinery isolated from these cell lines. One of the notable conclusions from this study was that all four ALS-causative proteins are required for interaction of the SMA-causative ASC-1 complex with RNAP II (BC *et al*., submitted). The observation that two different components of the ASC-1 complex are mutated to cause SMA and that the ALS-causative proteins mediate the association of the ASC-1 complex with RNAP II provide excellent examples of the importance of identifying interaction partners of ALS/SMA-causative proteins, as these interaction partners themselves are candidates for causing the diseases. In addition, identification of their interaction partners will assist in identifying molecular pathways involved in the pathogenesis of motor neuron disease.

In the present study, we report the interactomes of FUS, EWSR1, TAF15 and MATR3, and show that all four of these proteins associate with U1 snRNP. Unexpectedly, comparison of the interactome of the U1 snRNP machinery with that of the RNAP II machinery shows that virtually the entire U1 snRNP machinery overlaps with the RNAP II machinery. Among the proteins present in the U1 snRNP/RNAP II machinery are multiple ALS/SMA-causative proteins. These data raise the possibility that the RNAP II/U1 snRNP machinery and the pathways in which it functions may underlie the pathogenesis caused by a host of motor neuron disease-causative proteins.

## Results and Discussion

### FUS, EWSR1, TAF15 and MATR3 associate with U1 snRNP

**Figure 1:**
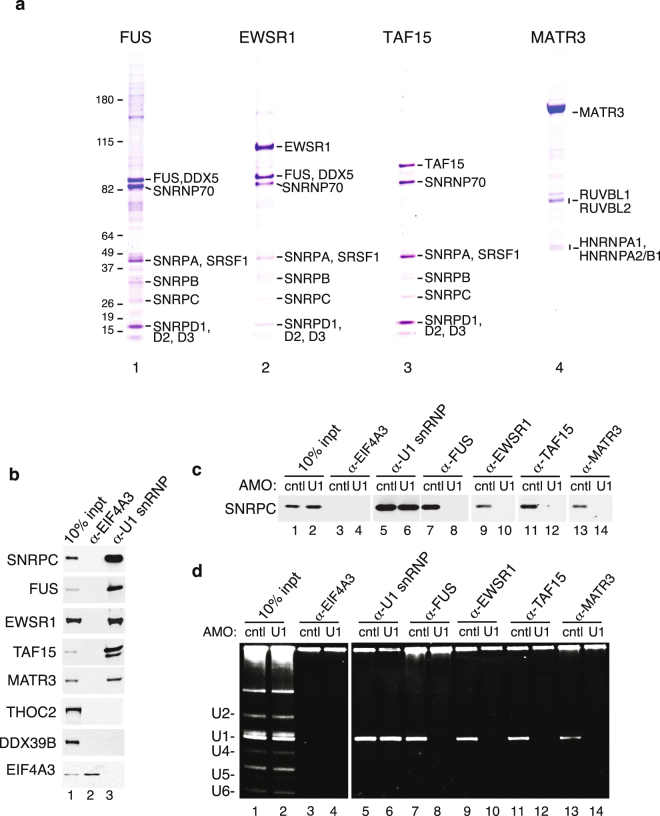
FET proteins and MATR3 associate with U1 snRNP. (**a**) Immunoprecipitations (IPs) were carried out with antibodies to FET proteins or MATR3 followed by analysis on a Coomassie-stained gel. Molecular weight markers and protein identified by mass spectrometry are indicated. (**b**) IPs were carried out from nuclear extract using a negative control antibody (EIF4A3) or an antibody to the SNRPC subunit of the U1 snRNP followed by Westerns with the indicated antibodies. (**c**) IPs were carried out with the indicated antibodies from nuclear extract treated with a U1 snRNA AMO or a negative control AMO followed by Western using the SNRPC antibody. (**d**) Same as (**c**) except that total RNAs from the IPs were examined on a denaturing gel stained with ethidium bromide.

To characterize the interactomes of FUS, EWSR1, TAF15 and MATR3 (hereafter referred to as ALS proteins) we immunopurified (IP’d) these proteins from HeLa cell nuclear extracts. To identify the highly abundant interactors, we excised individual bands from a Coomassie-stained gel and carried out mass spectrometry. This analysis revealed that U1 snRNP components are enriched in the FUS, EWSR1 and TAF15 IPs. These components include all of the U1 snRNP-specific proteins (SNRNP70, SNRPA, SNRPC) as well as the snRNP core proteins (SNRP proteins) (Fig. 1a, lanes 1–3). U1 snRNP components were not observed in the MATR3 IP (Fig. 1a, lane 4). We next carried out reciprocal IP/westerns using an antibody against the SNRPC core component of U1 snRNP. As shown in Fig. 1b, the three FET family members and MATR3 co-IP’d with U1 snRNP, but not with the negative control nuclear protein EIF4A3. Although U1 components were not detected on the Coomassie gel in the MATR3 IP, this may be due to a buried epitope (see below for mass spectrometry data of the U1 snRNP machinery that support this conclusion).

To determine whether the association between the ALS proteins and U1 snRNP was specific, we treated nuclear extracts with an anti-sense morpholino (AMO) that binds to the 5’ end of U1 snRNA and blocks splicing^27^. This U1 AMO also disrupted the association of FUS with U1 snRNP^28^. We obtained the same results in the present study (Fig. 1c, lanes 5–8). In addition, the U1 AMO disrupted the interactions between U1 snRNP and TAF15, EWSR1 and MATR3 (Fig. 1c, lanes 9–14). We further confirmed these associations by carrying out IPs and analyzing total RNA on an ethidium bromide stained gel. As shown in Fig. 1d, all of the ALS proteins co-IP’d with U1 snRNA, and the interaction was specific as it was disrupted by the U1 AMO. We conclude that FUS, TAF15, EWSR1 and MATR3 associate with U1 snRNP.

**Figure 2:**
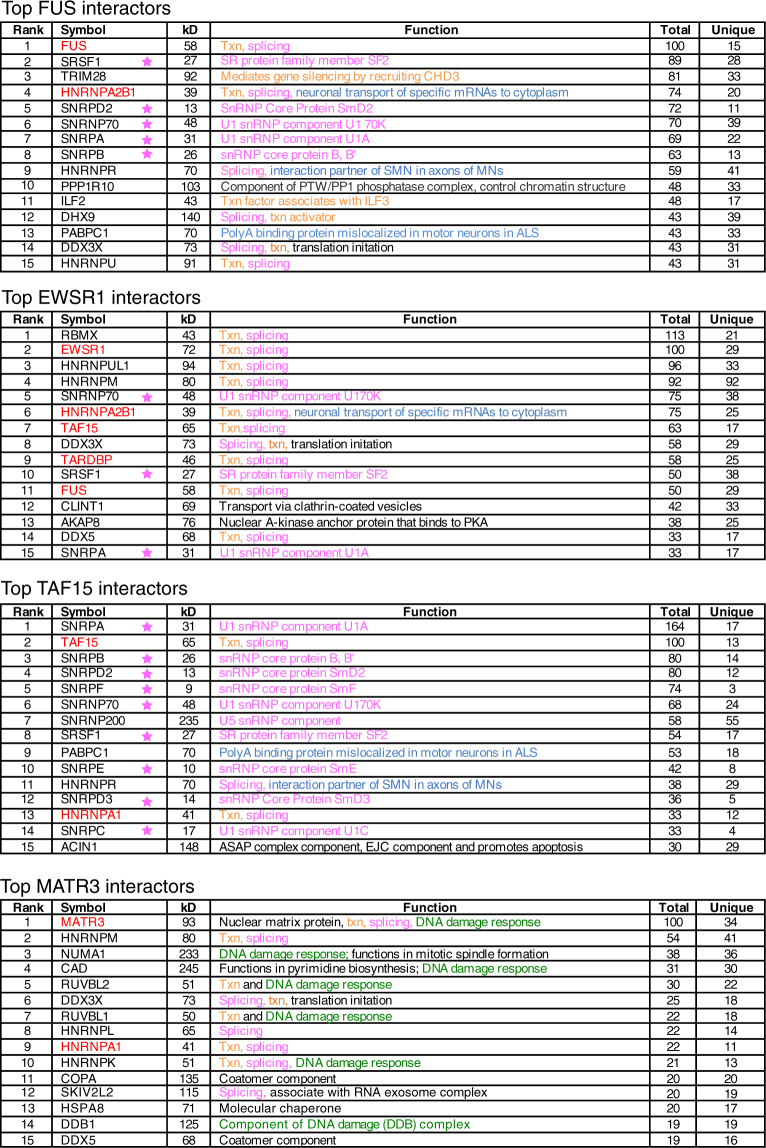
Top hit interactors in the FET proteins and MATR3 interactomes. The top ranked (by total peptide number) proteins in each interactome are shown. The rank, HGNC official symbol, calculated molecular weight, best-known function, total and unique peptide counts are shown. Functions in splicing (pink), transcription (txn, orange), DNA damage response (green), neuronal (blue) and other (black) are indicated. The symbols of ALS-causative proteins are in red. The stars indicate U1 snRNP components.

### FUS, EWSR1, TAF15 and MATR3 interactomes

To gain further insight into the interactomes of the four ALS-causative proteins, we carried out shotgun mass spectrometry of each IP. These data revealed 156, 68, 132 and 91 interactors for FUS, EWSR1, TAF15 and MATR3, respectively (Table S1). We listed the well-known functions and/or functions potentially relevant to motor neuron disease in the table for all of the interactors (color coded in Table S1). The top 15 hits in each interactome are shown in Fig. 2. Consistent with the Coomassie gel, canonical U1 snRNP components are among the top hits in the FUS, EWSR1 and TAF15 interactomes (marked by stars). In the EWSR1 interactome, multiple ALS-causative proteins are present (color coded red, Fig. 2). A top interactor of TAF15 is PABPC1, which is known to be mislocalized in ALS patient motor neurons^29^. A top hit in both the FUS and TAF15 interactomes is HNRNPR, which interacts with SMN1 in the axons of motor neurons^30,31^. HNRNPR is also found in the EWSR1 and MATR3 interactomes (ranked 34 and 19, respectively in Table S1). The observation that HNRNPR is a common interactor of ALS proteins and SMN1 reveals a new molecular link between ALS and SMA. Among the top hits in the MATR3 interactome are numerous proteins that function in the DNA damage response, including NUMA1, CAD, RUVBL1, RUVBL2, HNRNPK and DDB1. Consistent with these results, MATR3 itself is involved in the DNA damage response^32^, which has emerged as a pathway disrupted in multiple types of ALS and SMA^33–39^. Moreover, both RUVBL1 and RUVBL2 are components of the HSP90/R2TP chaperone complex, which interacts with the SMN complex and functions in facilitating snRNP assembly^40,41^, suggesting an involvement of MATR3 in snRNP assembly.

**Figure 3:**
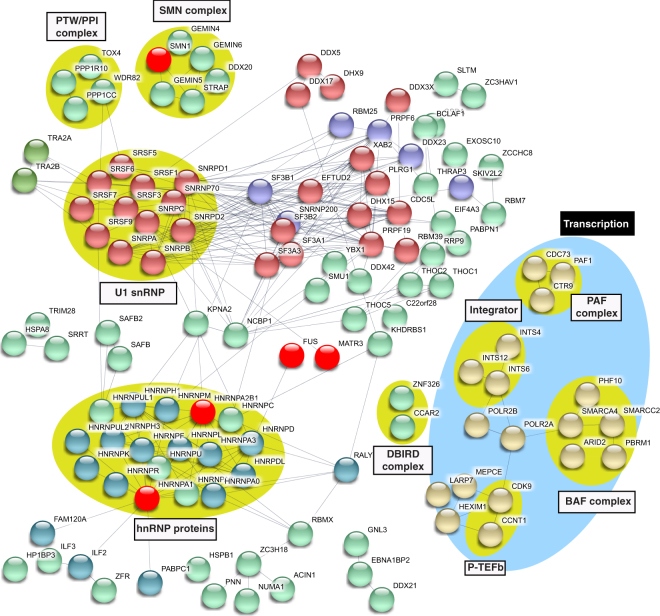
Protein-protein interaction network of the FUS interactome. The network of the FUS interactome constructed using STRING database (confidence score >0.7) is shown. Disconnected nodes are omitted. Protein complexes and clusters of functionally related proteins are indicated by yellow circles and blue circles, respectively. The ALS and SMA-causative proteins are in red.

**Figure 4:**
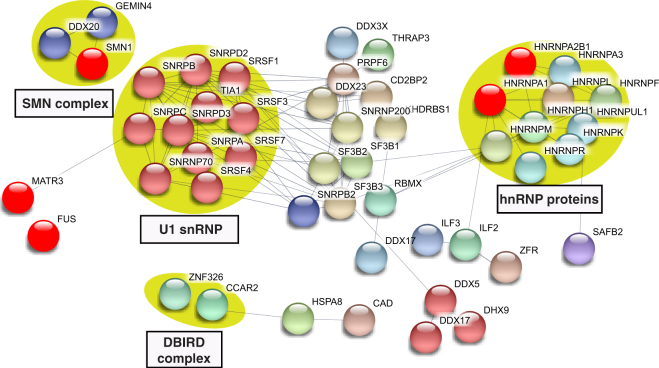
Protein-protein interaction network of the EWSR1 interactome. Same as Fig. 3, except that the network of the EWSR1 interactome is shown. The ALS and SMA causative proteins are in red.

**Figure 5:**
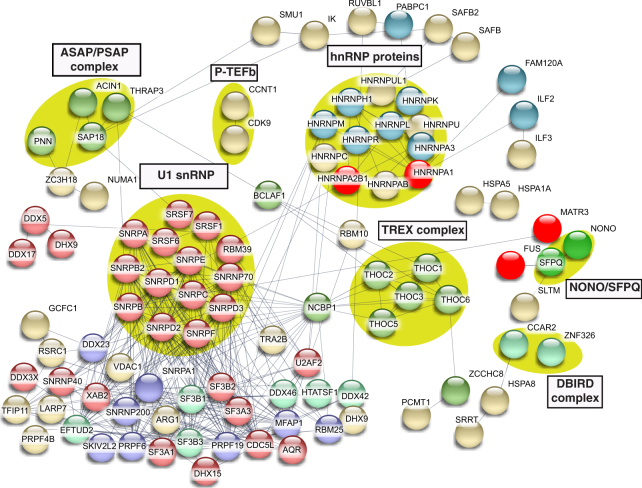
Protein-protein interaction network of the TAF15 interactome. Same as Fig. 3, except that the network of the TAF15 interactome is shown. The ALS causative proteins are in red.

**Figure 6:**
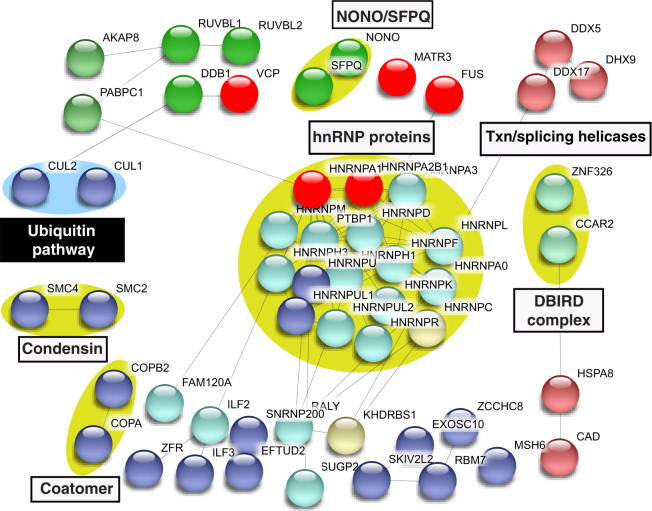
Protein-protein interaction network of the MATR3 interactome. Same as Fig. 3, except that the network of the MATR3 interactome is shown. The ALS causative proteins are in red.

To identify complexes in each of the interactomes, we analyzed the data in Table S1 using the STRING database (https://string-db.org). This analysis showed that each of the interactomes contain numerous distinct complexes. Several well-known complexes were not separated into clusters by STRING. Thus, we manually clustered these proteins (Figs 3–6). These data revealed complexes in common among the ALS protein interactomes so we next analyzed the proteins shared by all four ALS interactomes (Fig. S1). These interactomes share three dead box helicases (DHX9, DDX5 and DDX17), which, similar to the ALS proteins, are DNA/RNA binding proteins with roles in transcription and splicing. The DBIRD complex, reported to function in coupling transcription to alternative splicing^42^, as well as hnRNP proteins, are also shared by the four interactomes. The factors that are shared by the 4 ALS protein interactomes are good candidates for being disease-relevant. Consistent with this possibility, several ALS-causative proteins are also shared among the 4 interactomes, including HNRNPA1, HNRNPA2B1, FUS and MATR3.

The data in Figs 3–6 reveal complexes/factors unique to one or more of the ALS protein interactomes. FUS and TAF15 interactomes contain the SMN complex, thereby linking both of these ALS proteins to the SMA-causative SMN1 protein (Figs 3 and 5). The FUS and TAF15 interactomes also contain the transcription elongation factor P-TEFb, and the FUS interactome uniquely contains many other factors/complexes that function in transcription, including subunits of RNAP II, and BAF, PAF and integrator complex components (Fig. 3). The TAF15 interactome also contains the TREX mRNA export complex, which has been tied to ALS previously^43–46^. In addition, ASAP RNA processing/apoptosis complex is present in the TAF15 interactome, and apoptosis is a pathway associated with ALS (Fig. 5)^47,48^. Finally, the MATR3 interactome contains CUL1 and CUL2, factors that are components of the ubiquitin proteasome pathway. This pathway is known to be important in ALS via genes such as UBQLN2, which is mutated to cause the disease (Fig. 6)^49,50^. We conclude that the four ALS-causative proteins have multiple interaction partners, many of which are linked to different pathways involved in ALS/SMA, and these interaction partners are new candidates for factors involved in pathogenesis via these known pathways. Moreover, on a more basic science note, our data reveal that, despite the structural similarities and their common association with U1 snRNP, the four ALS proteins have many unique interaction partners that likely explain the distinct roles that these proteins have been reported to play in such processes as transcription and splicing.

### The U1 snRNP machinery overlaps extensively with the RNAP II machinery

**Figure 7:**
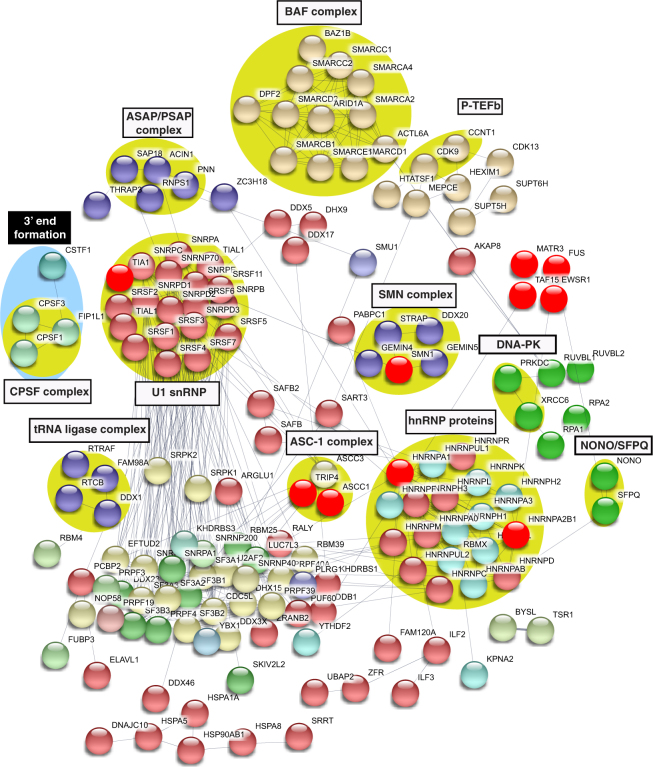
Protein-protein interaction network of the U1 snRNP machinery. Same as Fig. 3, except that the network of the U1 snRNP interactome is shown.

**Figure 8:**
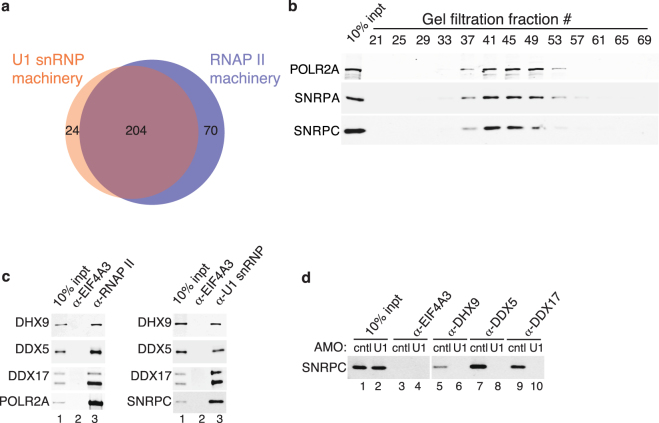
The U1 snRNP machinery overlaps with the RNAP II machinery. (**a**) Venn diagram showing overlap of the U1 snRNP and RNAP II machineries. (**b**) HeLa cell nuclear extract was separated on a Sephacryl-S500 column. The indicated fractions were used for Western analyses with antibodies against RNAP II and U1 snRNP components (SNRPA and SNRPC). Fraction 25 is the void volume and 69 is the included volume. (**c**) IPs were carried out from nuclear extract using an antibody to the POLR2A subunit of the RNAP II (left panel) or an antibody to the SNRPC subunit of the U1 snRNP (right panel) as well as a negative control antibody (EIF4A3) followed by Westerns with antibodies to the DEAD box helicases (DHX9, DDX5 and DDX17). (**d**) IPs were carried out with the indicated antibodies from nuclear extract treated with a U1 snRNA AMO or a negative control AMO followed by Western using the SNRPC antibody.

In light of the observation that FUS, EWSR1, TAF15 and MATR3 all associate with U1 snRNP, we next investigated the interactome of this machinery, identifying 226 proteins within it. These proteins and their functions are color coded in Table S2. As expected, the top two hits are core components (SNRNP70 and SNRPA) of U1 snRNP and the other core component (SNRPC), which is low molecular weight, is 48^th^ on the list. Numerous SRSFs and the snRNP core proteins (SNRPs) that are known U1 snRNP components are also in the interactome. We next investigated the U1 interactome using STRING (Fig. 7). Unexpectedly, this analysis revealed numerous complexes not typically associated with the role of U1 snRNP as a canonical splicing factor. Indeed, the U1 snRNP interactome contained many complexes that we recently identified in the interactome of the RNAP II machinery. The latter machinery contains 274 proteins (BC *et al*., submitted). Thus, we next directly compared the U1 snRNP and RNAP II machineries to one another. Remarkably, as shown in the Venn diagram, we found that virtually the entire U1 snRNP machinery interactome (>90%) overlaps with the RNAP II machinery interactome (Fig. 8a). The extent of the overlap between the U1 snRNP and RNAP II machineries is exemplified by the observation that core U1 snRNP components (SNRNP70, SNRPC, SNRNA) and SNRP core proteins are among the most abundant components of both the U1 snRNP and RNAP II machineries (Table S2 and BC *et al*., submitted). The abundance of the U1 snRNP components is readily apparent on a Commassie stained gel in which high levels of these components can be seen in both the U1 snRNP and RNAP II machineries (Fig. S2). Moreover, consistent with our observation that that the two machineries overlap, we found that RNAP II elutes in the same fractions as U1 snRNP components (SNRPA and SNRPC) in the high molecular weight region of a gel filtration column (Fig. 8b).

To validate the association of components of the U1 snRNP machinery with the RNAP II machinery, we carried out IP/Westerns. As shown in Fig. 8c, three DEAD box helicases, DHX9, DDX5, and DDX17, which are shared by both machineries all co-IP with both RNAP II and U1 snRNP, but not with the negative control EIF4A3. In addition, reciprocal IPs showed that all three proteins co-IP with U1 snRNP, and this association is specific as it is completely disrupted by the U1 AMO (Fig. 8d). Although the U1 snRNP machinery is highly abundant in RNAP II IPs, we do not observe reciprocal IPs of the RNAP II machinery using antibodies against U1 snRNP. One possible explanation for this is that the epitope on U1 snRNP that is recognized by the antibody is buried within the RNAP II machinery. We note that antibodies against the two other U1 snRNP core components also do not co-IP the RNAP machinery.

In addition to the U1 snRNP components and the DEAD box helicases, the two machineries have numerous complexes in common. Included among these are transcription complexes (P-TEFb, the BAF complex, and the ASC-1 complex), the SMN complex, the tRNA ligase complex, the NONO-SFPQ complex, the DNA-PK complex and the ASAP complex. The RNAP II and U1 snRNP machineries also share numerous proteins that are ALS or SMA causative. Both the ASC-1 complex and SMN complex contain SMA-causative proteins (ASCC1 and TRIP4, and SMN1, respectively). The other shared SMN-causative proteins are HSPB1 and EXOSC8. Multiple ALS-causative proteins are also in common, including FUS, EWSR1, TAF15, MATR3, TIA1, HNRNPA1, and HNRNPA2B1. Thus, the RNAP II/U1 snRNP machinery is clearly extensively associated with motor neuron disease-causative proteins.

There are 24 proteins unique to the U1 snRNP interactome. Among these are key components of the 3’ end formation machinery, including CPSF1, which binds to the AAUAAA polyadenylation signal and CPSF3, which is the endonuclease that cleaves the transcript prior to addition of the polyA tail^51–53^. Previous studies showed that the binding of U1 snRNP to 5’ splice sites blocks 3’ end formation at cryptic polyA sites in a process known as telescripting^54^. The mechanisms behind this are not understood. Our observation that critical 3’ end formation factors associate with U1 snRNP raises the possibility that these factors are the targets for U1 snRNP during telescripting.

70 proteins are specific to the RNAP II machinery, including the subunits of RNAP II itself as well as general transcription factors (e.g. TFIIF and NELF complex). It is not clear why some transcription factors are shared by the U1 snRNP and RNAP II machineries, whereas others are specific to RNAP II. One possibility is that the shared transcription factors are involved in coupling between transcription and splicing. It is well known that transcription by RNAP II potently enhances splicing, and our previous work indicated that this enhancement is due to the association of U1 snRNP with the RNAP II machinery, which allows efficient recruitment of U1 snRNP to 5’ splice sites during pre-mRNA synthesis^28,55,56^. There is also evidence that reciprocal coupling occurs in which splicing enhances transcription, but the mechanisms involved in this coupling are less well understood^57^. Our observation that the U1 snRNP machinery associates extensively with transcription factors and with the RNAP II machinery suggests that these interactions are involved in the reciprocal coupling. In particular, HTATSF1, which associates with p-TEFb, was previously identified as a factor involved in the reciprocal coupling^57^, and both factors are present in the U1 snRNP and RNAP II machinery interactomes. Thus, the association between the RNAP II and U1 snRNP machinery may be the molecular mechanism for bi-directional coupling between transcription and splicing.

As mentioned above, the RNAP II/U1 snRNP machinery contains numerous motor-neuron disease causative proteins and thus the processes in which the RNAP II/U1 snRNP machinery functions are candidates for pathways involved in the pathogenesis of motor neuron disease. These pathways include transcription, splicing, reciprocal coupling of transcription and splicing, snRNP biogenesis and DNA repair. In addition, the unexpected association of other factors, such as the ASAP complex and the tRNA ligase complex with the RNAP II/U1 snRNP machinery raises the possibility that other pathways are involved in the pathogenesis of ALS/SMA.

## Material and Methods

### Plasmids and Antibodies

The monoclonal antibodies used in this study were 8WG16 (against POLR2A, the large subunit of RNAP II) from Biolegend (cat # 920102), SNRPC from Sigma (cat # SAB4200188), DHX9 from Abcam (cat # ab26271), SNRPA (cat # sc-101149), DDX5 (cat # sc-166167), DDX17 (cat # sc-86409) from Santa Cruz. The polyclonal antibodies were FUS (cat # A300–293A), EWSR1 (cat # A300–418A) and MATR3 (cat # A300–591A) from Bethyl, TAF15 from Novus (cat # NB100–567), DDX17 (cat # sc-86409) from Santa Cruz, and DHX9 (cat # ab 26271) from Abcam. Our rabbit polyclonal antibodies to THOC2, EIF4A3, and DDX39B have been described^58,59^.

### Immunoprecipitations (IPs)

For IPs, antibodies were coupled to Protein A Sepharose beads (GE healthcare) and covalently cross-linked using dimethylpimelimidate (Sigma). Reaction mixtures (1 ml) contains 300 μl of HeLa nuclear extract^60^, 300 μl of SDB (20 mM HEPES, pH 7.9, 100 mM KCl), 500 μM ATP, 3.2 mM MgCl_2_ and 20 mM creatine phosphate. The mixtures were incubated for 30 min at 30 °C to turn over endogenous complexes in the nuclear extract^56^. Reaction mixtures were then added to 500 μl of buffer A (1X PBS, 0.1% Triton, 0.2 mM PMSF, protease inhibitor EDTA-free [Roche]) and 40 μl of antibody-crosslinked beads. The IPs were carried out overnight at 4 °C. After five washes with buffer A, proteins were eluted at room temperature using 80 μl of protein gel loading buffer (125 mM Tris, 5% SDS, 20% glycerol, 0.005% Bromophenol blue). After elution, DTT was added to a final concentration of 40 mM, and 15 μl of each eluate was analyzed on a 4–12% SDS-PAGE gradient gel (Life technologies). AMO treatment was performed by adding control AMO (5′-CCTCTTACCTCAGTTACAATTTATA-3′) or U1 AMO (5′-GGTATCTCCCCTGCCAGGTAAGTAT-3′)^27,28^ to HeLa nuclear extract to a final concentration of 12 μM before IP. For analysis of total RNAs in the IPs, beads were treated with proteinase K for 10 min at 37 °C, and RNAs were recovered by phenol/chloroform extraction and ethanol precipitation. RNAs were run on 8% denaturing polyacrylamide gels and stained with Ethidium Bromide.

### Mass Spectrometry

To identify the interactomes of FUS, EWSR1, TAF15 and MATR3, the IP samples were trichloroacetic acid (TCA) precipitated and subjected to shotgun mass spectrometry. The total peptide number of the antigen in each IP was set as 100 and the relative peptide numbers of each interactors are shown in Table S1. Abundant cytoplasmic proteins, ribosomal proteins, proteins greater than 250 kDa, and proteins for which the relative total peptide number is smaller than 5 were omitted. For mass spectrometry of the U1 snRNP machinery, the IP was TCA precipitated and the digested peptides were labeled by tandem mass tag^61^ for MS3 analysis on an Orbitrap Fusion mass spectrometer coupled to a Proxeon EASY-nLC 1000 liquid chromatography (LC) pump (Thermo Scientific). Abundant cytoplasmic proteins, ribosomal proteins, proteins greater than 200 kDa with less than 10 spectral counts, proteins greater than 70 kDa with less than 4 spectral counts, and proteins with one spectral count were not included in Table S2. The proteins in Tables S1 and S2 were annotated with functions using the Gene Cards database (www.genecards.org)^62^ and/or searching the literature. To compare the U1 snRNP machinery and the RNAP II machinery, the quantitative mass spectrometry data of the WT RNAP II machinery (BC *et al*., submitted) was filtered using the same criteria used for the U1 snRNP machinery as mentioned above.

### Gel filtration

A reaction mixture containing 300 μl of HeLa nuclear extract^60^, 300 μl of SDB (20 mM HEPES, pH 7.9, 100 mM KCl), 500 μM ATP, 3.2 mM MgCl_2_ and 20 mM creatine phosphate was incubated for 30 min at 30 °C. After incubation, the mixture was separated on a Sephacryl S500 (GE Healthcare) gel filtration column. The gel filtration column buffer contains 20 mM HEPES, 60 mM KCl, 2.5 mM EDTA and 0.1% Triton X-100.

### Data availability statement

The materials and datasets generated during and/or analyzed during the current study are available from the corresponding author upon request.

## Electronic supplementary material

Supplemental Table S1

Supplemental Table S2

Supplementary Information
