# Supplementary material for: Interactome analyses revealed that the U1 snRNP machinery overlaps extensively with the RNAP II machinery and contains multiple ALS/SMA-causative proteins
Source: Sci Rep. 2018 Jun 8;8:8755. doi: 10.1038/s41598-018-27136-3 (PMC5993797; doi:10.1038/s41598-018-27136-3)

Interactome analyses revealed that the U1 snRNP machinery overlaps extensively with the RNAP II machinery and contains multiple ALS/SMA-causative proteins

Binkai Chi[1], Jeremy D. O'Connell[1,2], Tomohiro Yamazaki[1], Jaya Gangopadhyay[1],

Steven P. Gygi[1] and Robin Reed[1]*

[1] Department of Cell Biology, Harvard Medical School,

240 Longwood Ave. Boston MA 02115

[2] Present Address: Department of Microbiology and Immunology, Stanford University School of Medicine,
291 Campus Drive Stanford, CA 94305

*corresponding author

e-mail: rreed@hms.harvard.edu

FAX (617) 432-3091

Phone (617) 432-1784

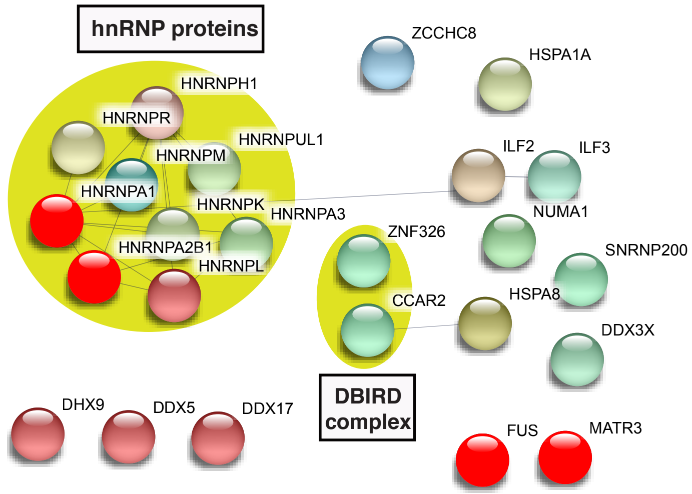

Figure S1. Shared interactos of FUS, EWSR1, TAF15 and MATR3. The PPI network of the interactors shared by four ALS proteins constructed using STRING database (confidence score >0.7) is shown. Protein complexes are indicated by yellow circles. The ALS-causative proteins are in red.

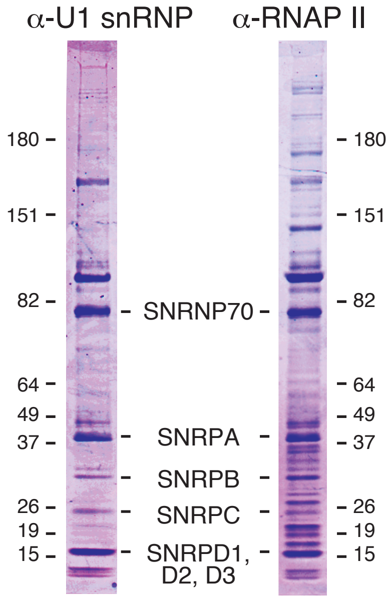

Figure S2. Canonical U1 snRNP proteins are shared components of the U1 snRNP and RNAP II machineriers. Immunoprecipitations (IPs) were carried out with the antibodies to U1 snRNP or RNAP II followed by analysis on a Coomassie-stained gel. Molecular weight markers and the canonical U1 snRNP proteins are indicated.

Full size original image of gel displayed in Fig 1a

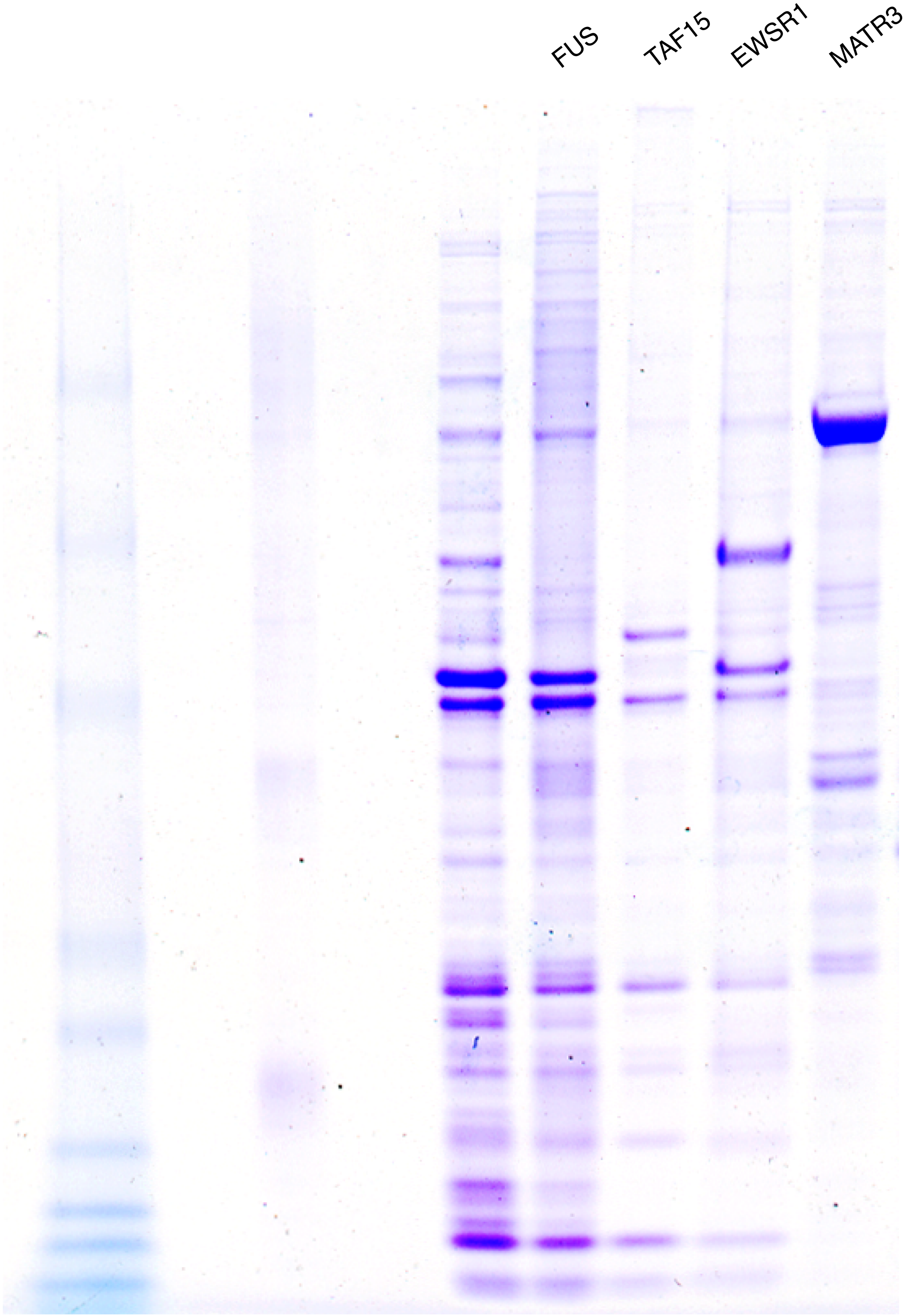

Full size original images of blots displayed in Fig 1b

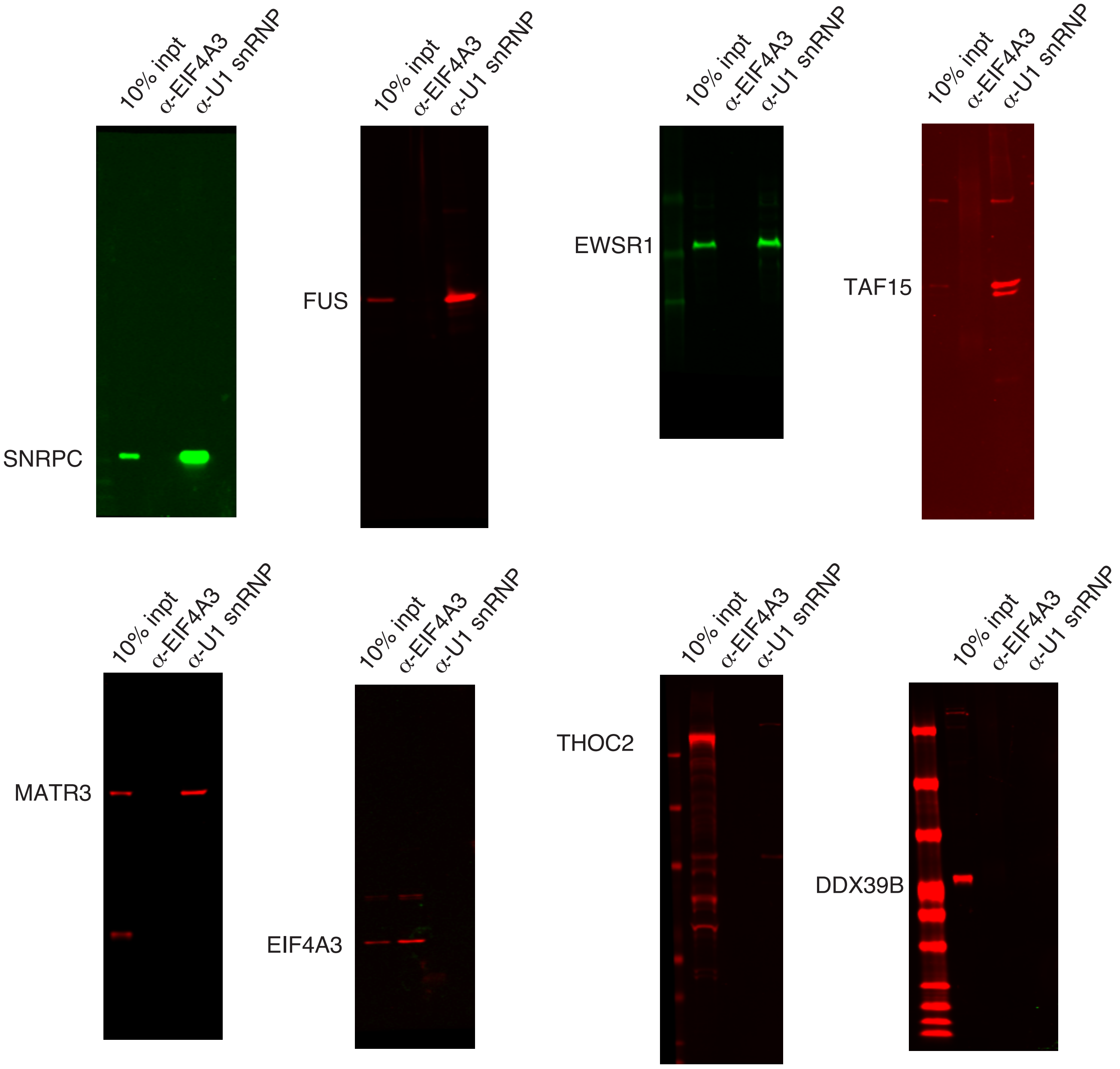

Full size images of blots displayed in Fig 1c

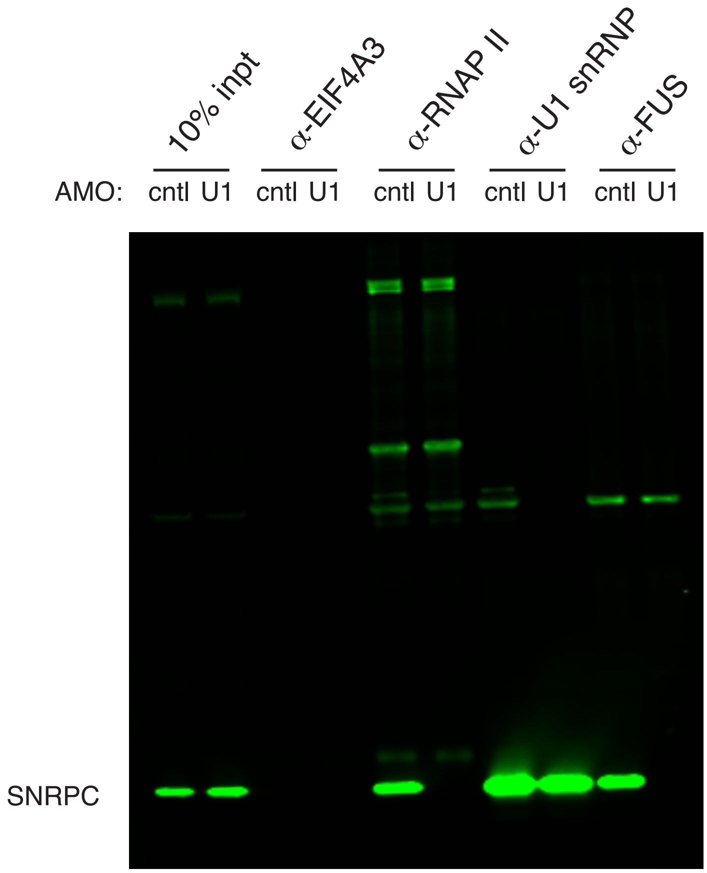

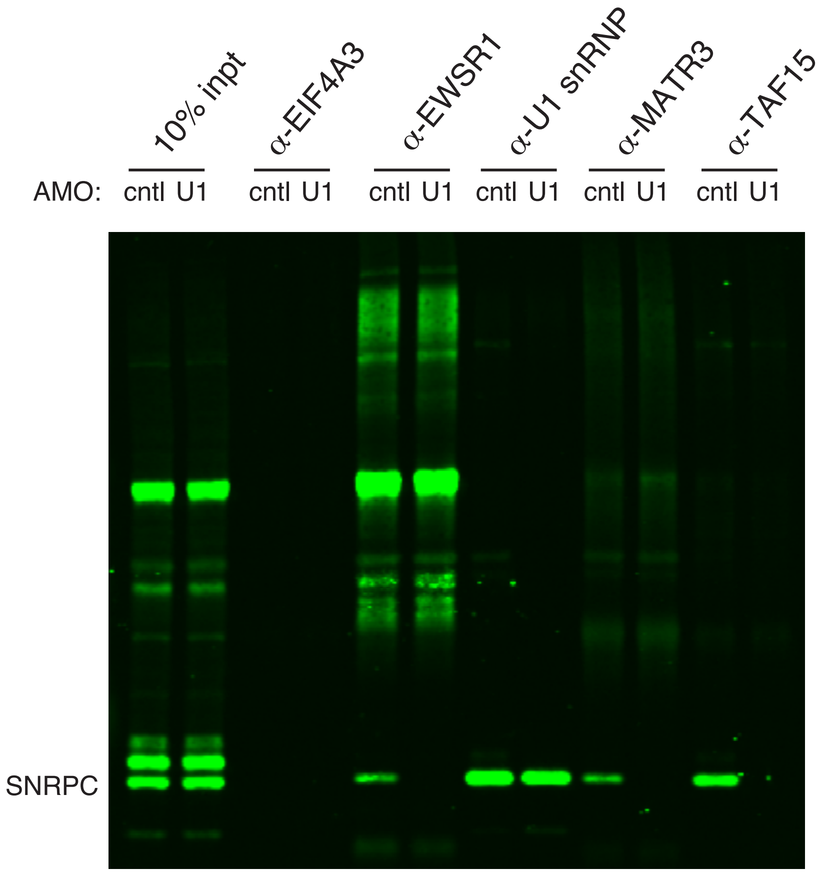

Full size original image of gel displayed in Fig 1d

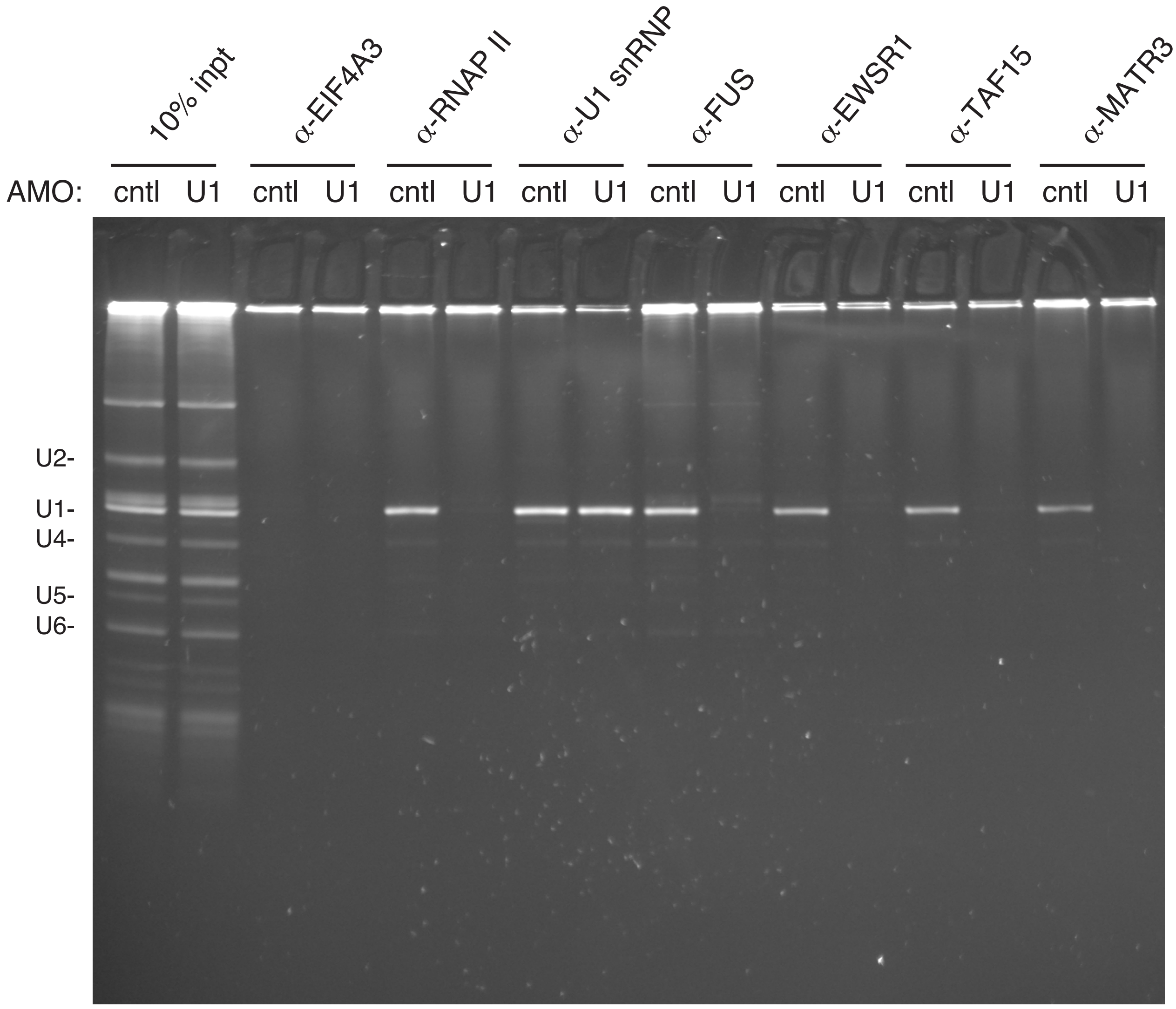

Full size original images of blots displayed in Fig 8b

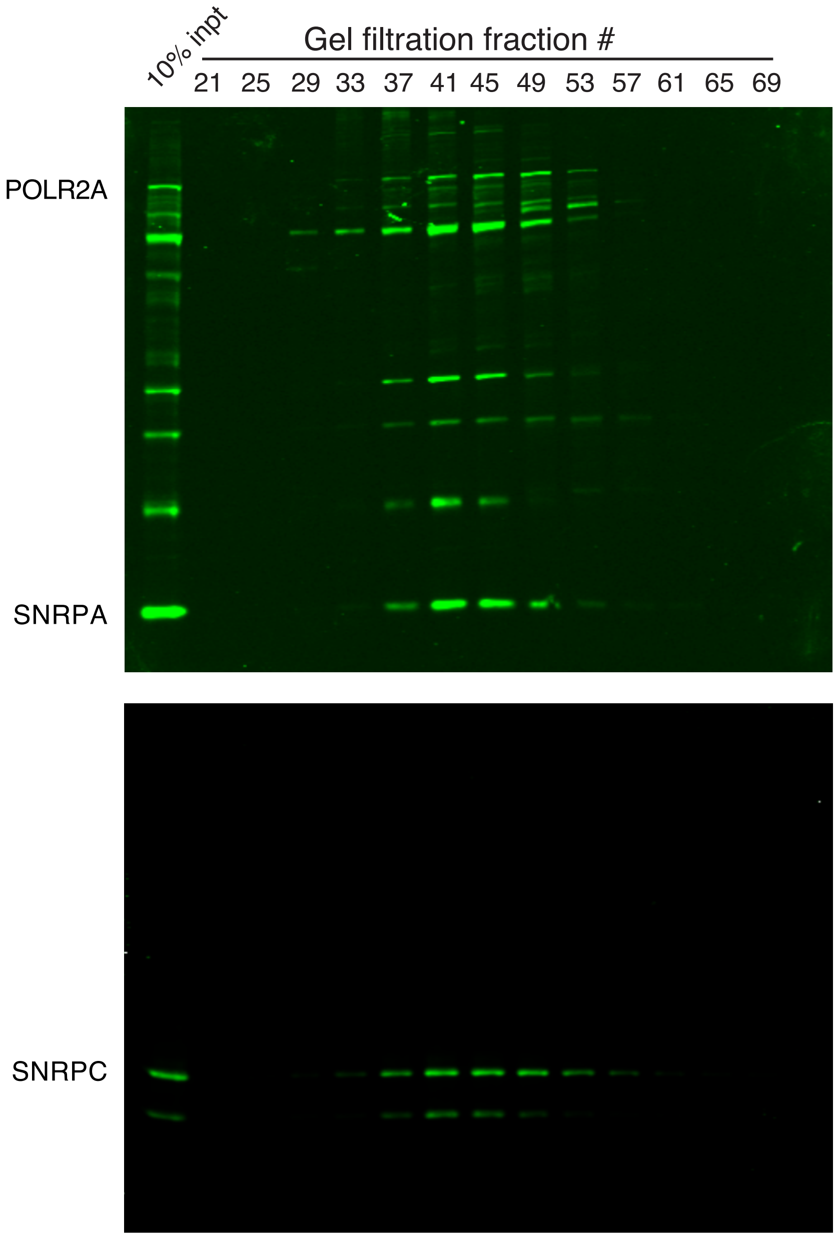

Full size original images of blots displayed in Fig 8c

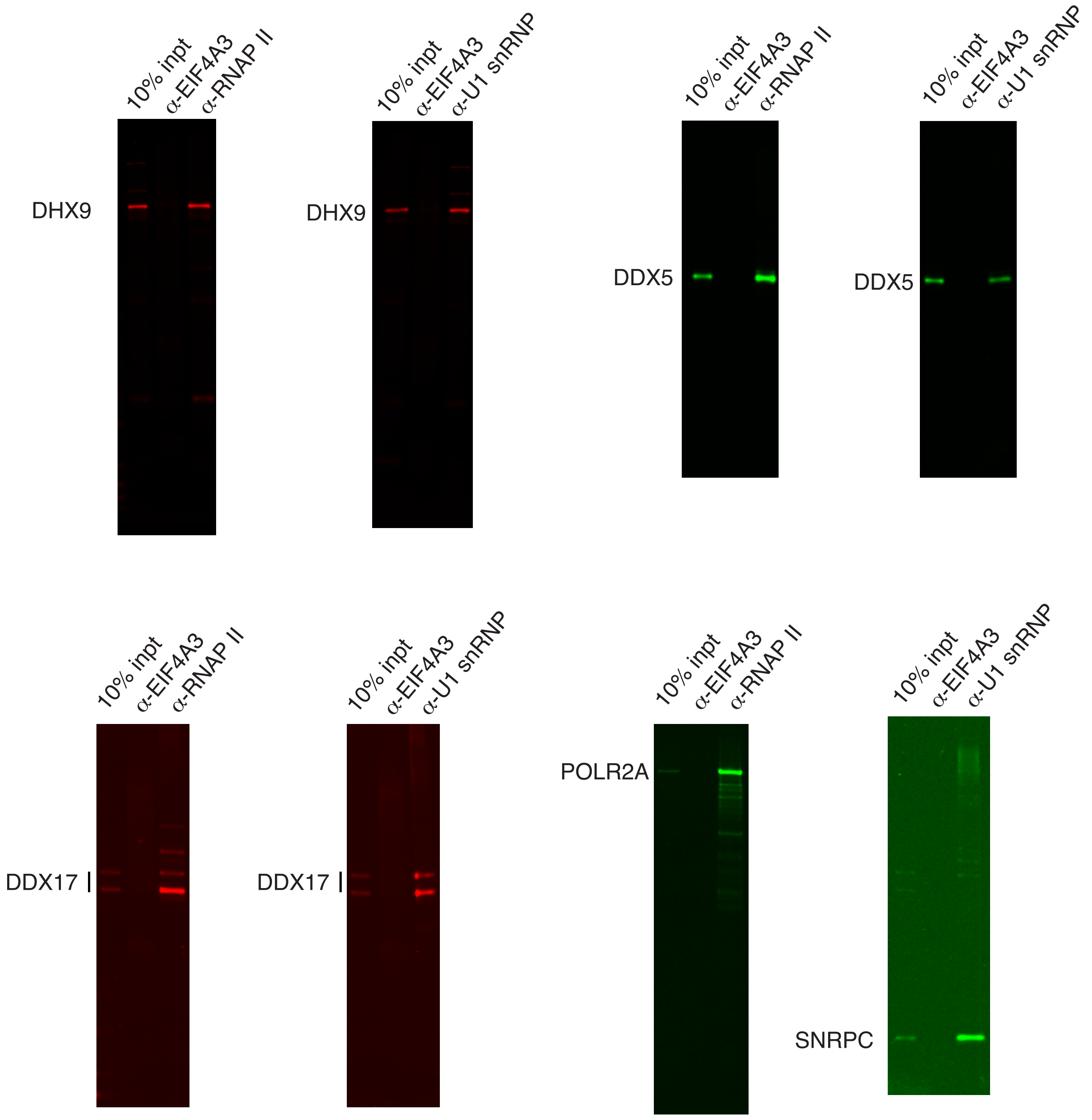

Full size original image of blots displayed in Fig 8d

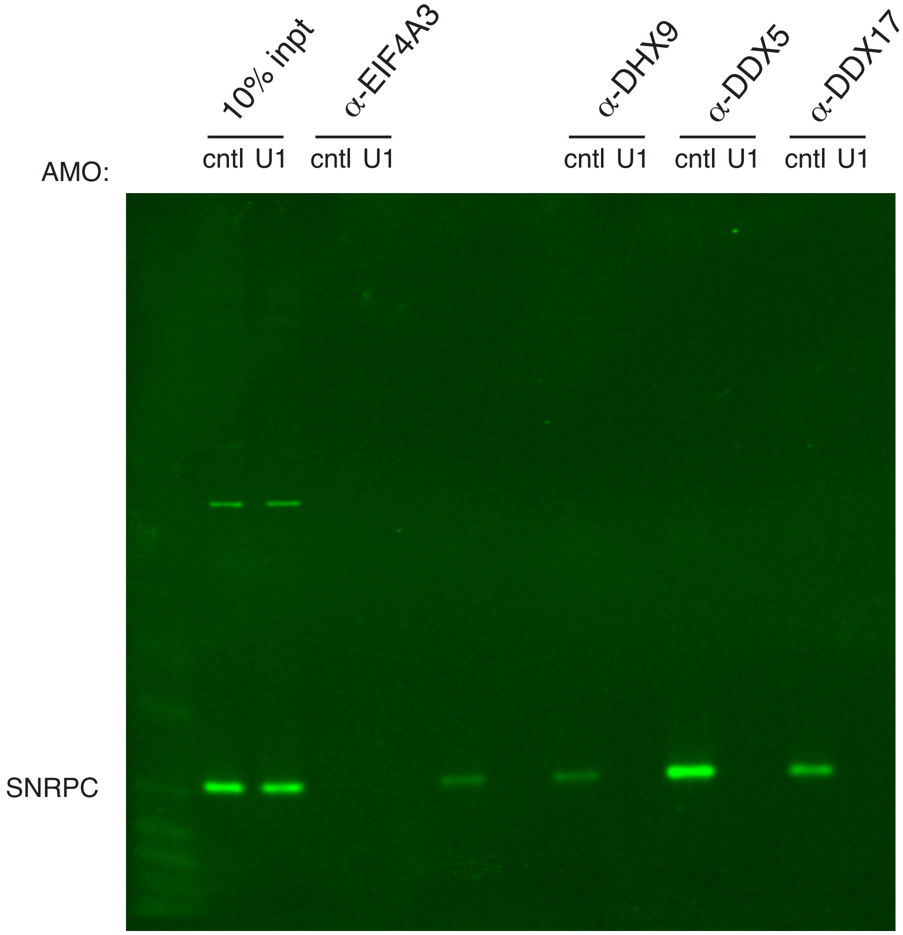

Full size original image of gel displayed in Fig S2

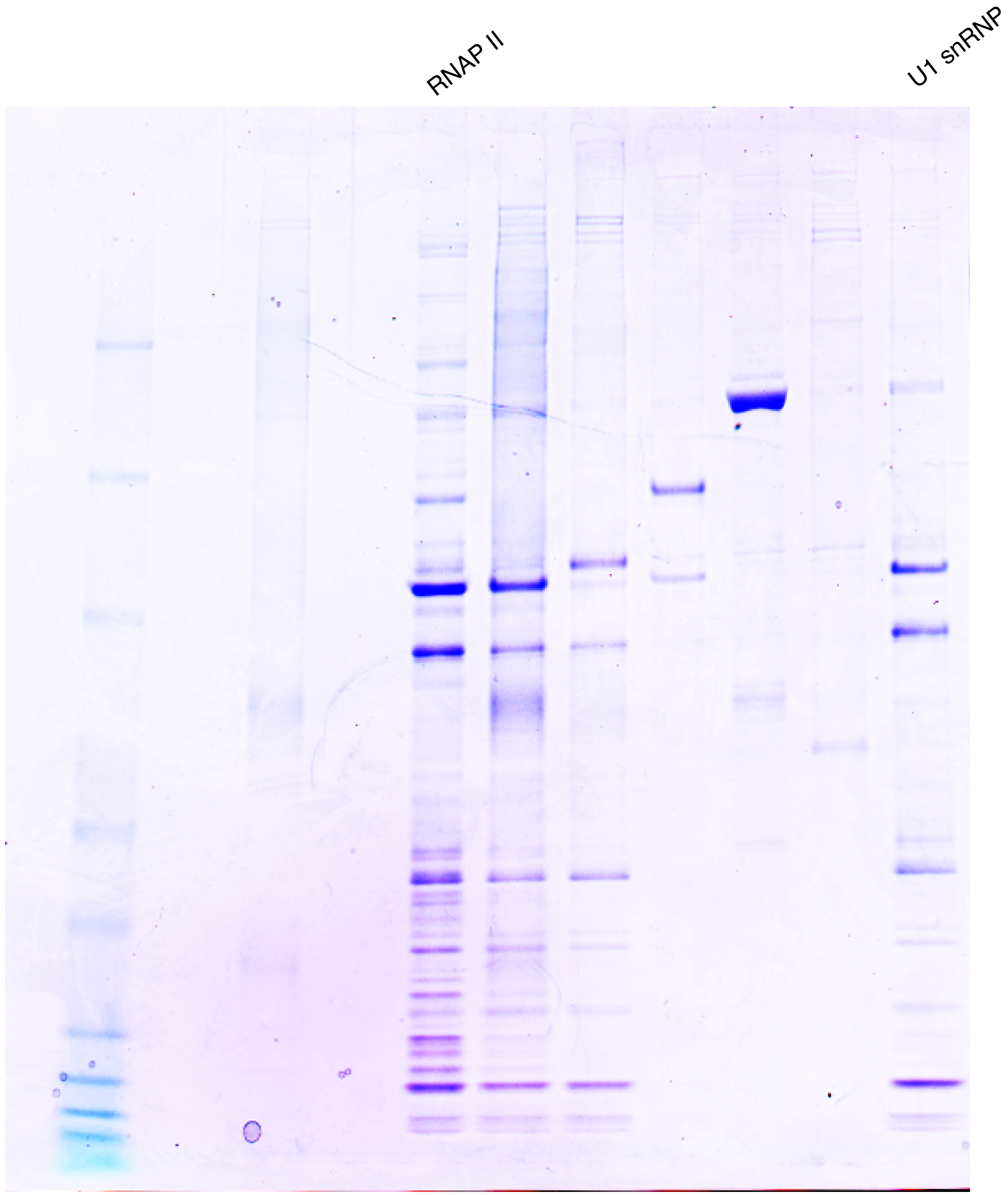

Supplement: Supplementary file 3 — Supplementary Information [file 41598_2018_27136_MOESM3_ESM.pdf]
